# Supplementary material for: Proteomic Analysis Reveals Key Proteins in Extracellular Vesicles Cargo Associated with Idiopathic Pulmonary Fibrosis In Vitro
Source: Biomedicines. 2021 Aug 20;9(8):1058. doi: 10.3390/biomedicines9081058 (PMC8394197; doi:10.3390/biomedicines9081058)
Supplement: Supplementary file 1 [file biomedicines-09-01058-s001.zip › additional information.pdf]

Proteomic analysis reveals key proteins in extracellular vesicles cargo associated with idiopathic pulmonary fibrosis in vitro

Juan Manuel Velázquez-Enríquez[1], Jovito Cesar Santos-Álvarez[1], Alma Aurora Ramírez-Hernández[1], Edilburga Reyes-Jiménez[1], Armando López-Martínez[1], Socorro Pina-Canseco[2], Sergio Roberto Aguilar-Ruiz[1], María de los Ángeles Romero-Tlalolini[5], Luis Castro-Sánchez[3], Jaime Arellanes-Robledo[4], Verónica Rocío Vásquez-Garzón[5] and Rafael Baltiérrez -Hoyos[5*]

[1] Facultad de Medicina y Cirugía, Universidad Autónoma Benito Juárez de Oaxaca, 68120, Oaxaca, México; juanmanuelvela_enriquez@live.com (J.M.V.-E.); jovitocesarsa@hotmail.com (J.C.S.-Á.); aramih_09@hotmail.com (A.A.R.-H.); edilreyesjimnez@yahoo.com.mx (E.R.-J.); armandoloopez37@gmail.com (A.L.-M.); sar_cinvestav@hotmail.com (S.R.A.-R.)

[2] Centro de Investigación, Facultad de Medicina, UNAM-UABJO, 68120, Oaxaca, Oax, México; socopina12@hotmail.com (S.P.-C.)

[3] CONACYT-Facultad de Medicina y Cirugía, Universidad Autónoma Benito Juárez de Oaxaca, 68120 Oaxaca, México; mdlaromerotl@conacyt.mx (M.Á.R.-T.) veronicavasgar@gmail.com (V.R.V.-G.)

[4] CONACYT-Universidad de Colima, Centro Universitario de Investigaciones Biomédicas "CUIB", Universidad de colima, 28045 Colima, Col, México; luis_castro@ucol.mx (L.C.-S.)

[5] CONACYT-Instituto Nacional de Medicina Genómica, 14610 Ciudad de México, México; jarellanes@inmegen.gob.mx (J.A.-R.)

* Correspondence: rbaltierrez@hotmail.com (R.B.-H.)

Supplementary Figures

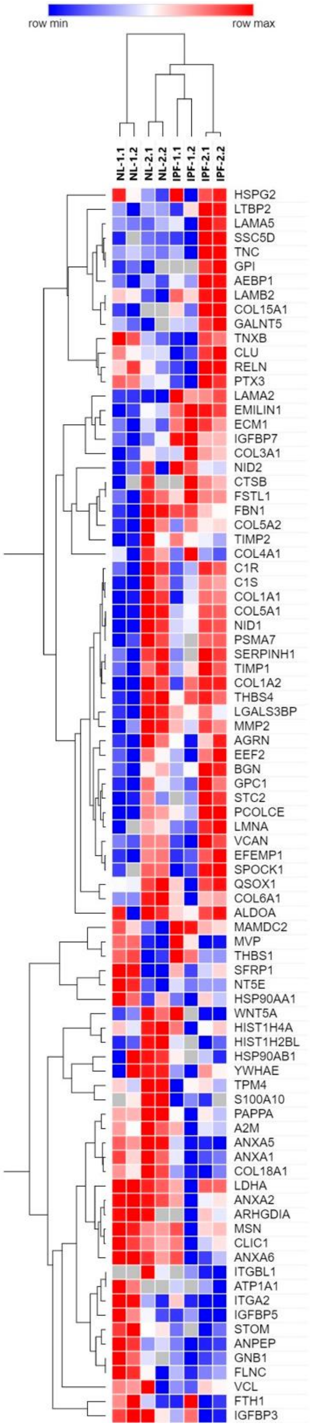

Figure S1: Heat map showing differentially expressed proteins in EVs cargo isolate from cell lines bearing both IPF and healty phenotype. Protein intensities were log2 transformed and Z-scored to be normalized between groups. Up-regulated and down-regulated proteins are depicted in red and blue colors, respectively. IPF, idiopathic pulmonary fibrosis; NL, normal lung.

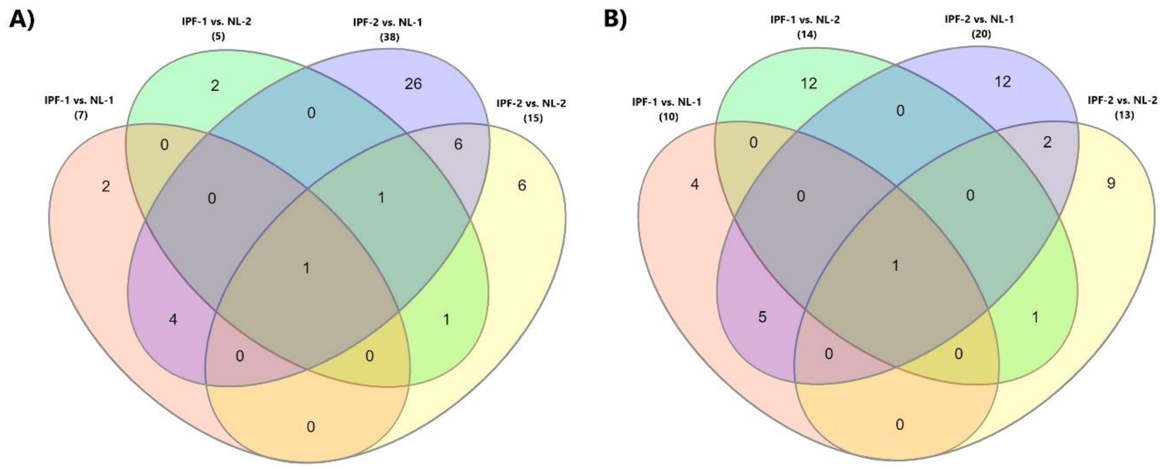

Figure S2: Distribution of differentially expressed proteins. (A) Venn diagram showing the distribution of up-regulated proteins in the comparison groups. (B) Venn diagram showing the distribution of down-regulated proteins in the comparison groups. IPF, idiopathic pulmonary fibrosis; NL, normal lung.

Supplementary Tables

Figure S1: Heat map showing differentially expressed proteins in EVs cargo isolated from cell lines bearing both IPF and healthy phenotype.

Figure S2: Distribution of differentially expressed proteins.

Table S1: Proteins identified by proteomic analysis using LFQ intensity value ≠ 0 in at least one biological replicate.

Table S2: Proteins identified by proteomic analysis using LFQ intensity value ≠ 0 and MS/MS (spectral) count ≥2 in at least one biological replicate.

Table S3: Proteins identified in a single EVs group.

Table S4: Proteins identified as differentially expressed (fold change ≥1 y ≤ −1 and $p < 0.05$) in IPF-1 vs. NL-1 comparison.

Table S5: Proteins identified as differentially expressed (fold change ≥1 y ≤ −1 and $p < 0.05$) in IPF-1 vs. NL-2 comparison.

Table S6: Proteins identified as differentially expressed (fold change ≥1 y ≤ −1 and $p < 0.05$) in IPF-2 vs. NL-1 comparison.

Table S7: Proteins identified as differentially expressed (fold change ≥1 y ≤ −1 and $p < 0.05$) in IPF-2 vs. NL-2 comparison.

Table S8: Proteins identified as differentially expressed (fold change ≥1 y ≤ −1 and $p < 0.05$) in two or more comparisons.

Table S9: GO enrichment (biological process) for up-regulated proteins in the pairwise comparisons using GeneCodis.

Table S10: GO enrichment (biological process) for down-regulated proteins in the pairwise comparisons GeneCodis.

Table S11: GO enrichment (molecular function) for up-regulated proteins in the pairwise comparisons using GeneCodis.

Table S12: GO enrichment (molecular function) for down-regulated proteins in the pairwise comparisons using GeneCodis.

Table S13: GO enrichment (cellular component) for up-regulated proteins in the pairwise comparisons using GeneCodis.

Table S14: GO enrichment (cellular component) for down-regulated proteins in the pairwise comparisons using GeneCodis

Table S15: Enrichment pathways (KEGG) for up-regulated proteins in the pairwise comparisons using GeneCodis.

Table S16: Enrichment pathways (KEGG) for down-regulated proteins in the pairwise comparisons using GeneCodis.
